# Advanced cutting strategy for navigated, robot-driven laser craniotomy for stereoelectroencephalography: An *in Vivo* non-recovery animal study

**DOI:** 10.3389/frobt.2022.997413

**Published:** 2022-09-12

**Authors:** Fabian Winter, Daniel Beer, Patrick Gono, Stefano Medagli, Marta Morawska, Christian Dorfer, Karl Roessler

**Affiliations:** ^1^ Department of Neurosurgery, Medical University of Vienna, Vienna, Austria; ^2^ Advanced Osteotomy Tools, Basel, Switzerland

**Keywords:** robotic, navigated, cutting strategy, SEEG, epilepsy surgery, depth electrodes

## Abstract

**Objectives:** In this study we aimed to present an updated cutting strategy and updated hardware for a new camera system that can increase cut-through detection using a cold ablation robot-guided laser osteotome.

**Methods:** We performed a preoperative computed tomography scan of each animal. The laser was mounted on a robotic arm and guided by a navigation system based on a tracking camera. Surgery was performed with animals in the prone position. A new cutting strategy was implemented consisting of two circular paths involving inner (full cylindric) and outer (hollow cylindric) sections, with three different ablation phases. The depth electrodes were inserted after cut-through detection was confirmed on either the coaxial camera system or optical coherence tomography signal.

**Results:** A total of 71 precision bone channels were cut in four pig specimens using a robot-guided laser. No signs of hemodynamic or respiratory irregularities were observed during anesthesia. All bone channels were created using the advanced cutting strategy. The new cutting strategy showed no irregularities in either cylindrical (parallel walled; *n* = 38, 45° = 10, 60° = 14, 90° = 14) or anticonical (walls widening by 2 degrees; *n* = 33, 45° = 11, 60° = 13, 90° = 9) bone channels. The entrance hole diameters ranged from 2.25–3.7 mm and the exit hole diameters ranged from 1.25 to 2.82 mm. Anchor bolts were successfully inserted in all bone channels. No unintended damage to the cortex was detected after laser guided craniotomy.

**Conclusion:** The new cutting strategy showed promising results in more than 70 precision angulated cylindrical and anti-conical bone channels in this large, *in vivo* non-recovery animal study. Our findings indicate that the coaxial camera system is feasible for cut-through detection.

## 1 Introduction

We recently introduced a navigated, robot-driven laser for implantation of cranial depth electrodes as an alternative to frame-based procedures (Spire et al., 2008; Cardinale et al., 2013; Berg et al., 2019; Roessler et al., 2021). This method was proven accurate and feasible in previous cadaver and *in vivo* non-recovery studies (Roessler et al., 2021; Winter et al., 2021). However, cut-through could not be reliably detected with neither optical coherence tomography (OCT) signals nor the coaxial camera system. Furthermore, our previous results were limited owing to the small sample size of 19 bone channels. The current study presents a novel cutting strategy as well as updated hardware in terms of a new camera system that increases cut-through detection. Therefore, we performed a large test series in this *in vivo* non-recovery study.

## 2 Materials and methods

This study was approved by the local Departement de Territori I Sostenibilitat (10076; FUE-2018-00726444 I ID KX68K1DZV). The surgeon performing the procedure was accredited with an EU Function A certificate for educational and training courses in laboratory animal science. The laboratory personnel were specifically trained in the use of pigs.

### 2.1 Pre- and intra-operative planning

**FIGURE 1:**
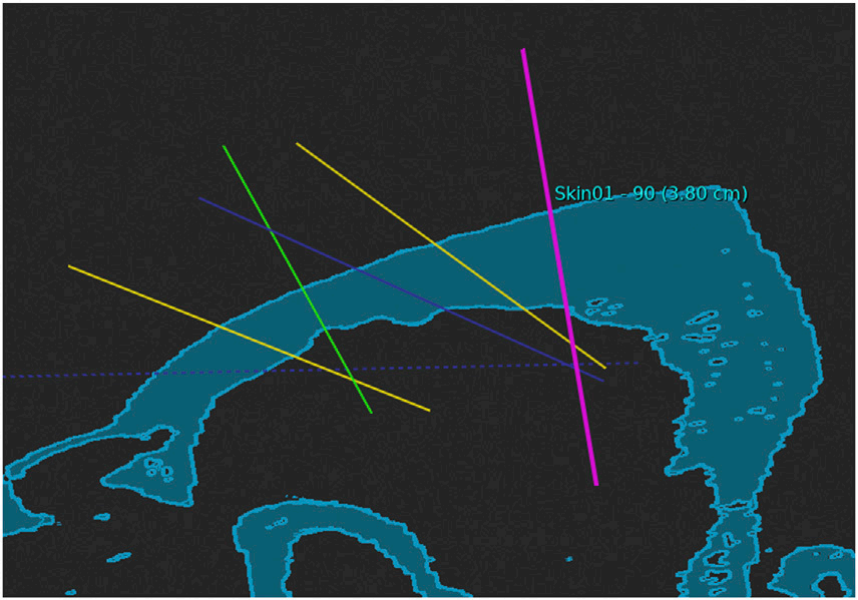
Planned trajectories for depth electrodes in a pig skull.

A computed tomography scan (Siemens Somatom Emotion 16 CT Scanner, Munich, Germany) was obtained of each head specimen with a 0.75-mm axial resolution. Digital Imaging and Communications in Medicine data was processed and validated into STL-files using surgical planning software (Neuro SEEG Planner, Advanced Osteotomy Tools AG, Basel, Switzerland). The data were transferred to the graphical user interface of the laser osteotome (CARLO^®^ primo+, Advanced Osteotomy Tools AG). Preoperative planning was performed to define the trajectories and target points for depth electrodes (Figure 1).

### 2.2 Anesthesia

This study was conducted in accordance with the European requirements (Directive EU/2010/63) and United States Food and Drug Administration Good Laboratory Practice regulations (21 CFR 58), and followed the test facility´s standards of care protocols. The pigs were sedated by intramuscular administration of dexmedetomidine (0.03 mg/kg), midazolam (0.3 mg/kg), and butorphanol (0.3 mg/kg) combined. Anesthesia was then induced with intravenous propofol (1 mg/kg); tracheal intubation was performed for mechanical ventilation. Isoflurane (2–4%) was used to maintain anesthesia throughout the procedure.

### 2.3 Registration procedure

The laser osteotome was mounted on a robotic arm (KUKA AG, Augsburg, Germany) that provides lateral repeatability of less than 0.15 mm and angular repeatability of less than 7 mrad. The robotic arm was designed to be tactile and to move at a safe speed and force, which facilitates human collaboration. The robot is guided by a navigation system that is based on a camera that tracks two marker sets: one integrated in the laser head and another attached to the specimen. Surgery was performed with pigs in a prone position and heads embedded in sand cushions. The pig’s position relative to the attached marker was determined by identifying five physical landmarks on the cranium for the registration procedure. Registration accuracy of less than 1.0 mm root mean square was considered acceptable.

### 2.4 Surgical procedure

**FIGURE 2:**
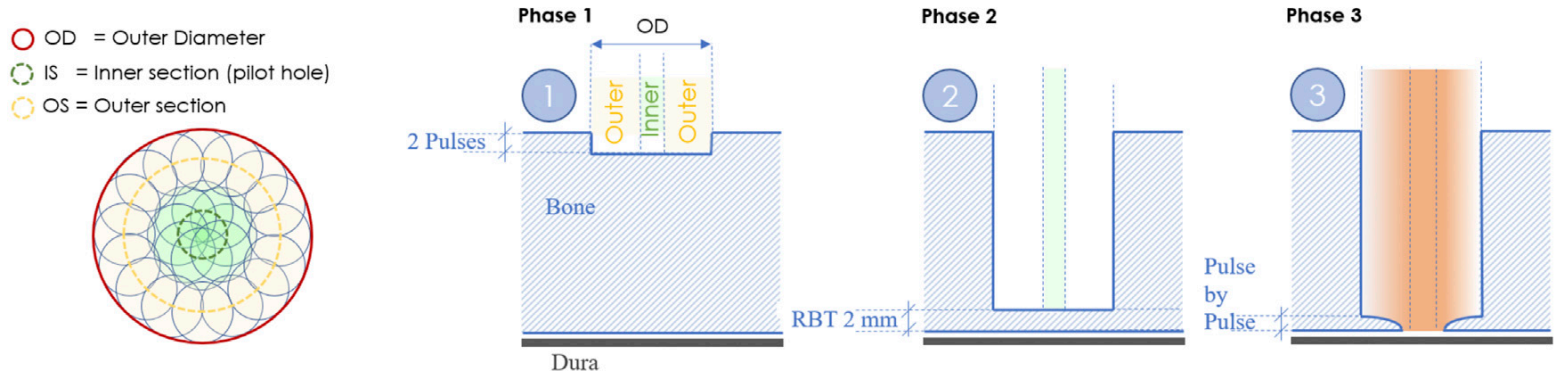
New cutting strategy for bone channels based on a centric pilot shot followed by a surrounding hollow-cylinder that shapes the contour of the hole.

The cold ablation robot-guided laser osteotome (CARLO^®^ primo+, Advanced Osteotomy Tools AG) uses a 2.94 μm erbium-doped yttrium aluminum garnet laser (Syneron Dental Laser, Yokneam, Israel) with an 0.8-mm focal diameter as previously described (Baek et al., 2015; Roessler et al., 2021, Winter et al., 2021). A new cutting strategy was implemented consisting of two circular paths, with inner and outer sections and three different ablation phases. Phase 1 consists of cutting the inner and outer sections with two pulses to initiate the OCT measurement. OCT is similar to ultrasonic imaging detection of the difference in acoustic waves using interferometry to produce images of optical light backscattering from microscopic inhomogeneous constituents within tissues (Yashin et al., 2017). This is followed by phase 2, which cuts the inner and outer sections with the remaining bone thickness using the OCT measurement. The outer wall can have a cylindrical (0°) or anti-conical design (−2°). Finally, phase 3 alternates between the inner and outer sections, pulse by pulse, to confirm cut-through (Figure 2). Cut-through or cerebral spinal fluid influx can be marked using the special button in the stereoelectroencephalography graphical user interface.

Once cut-through detection was confirmed on either the coaxial camera system or OCT signal, the depth electrodes were inserted to confirm the cut-through and trajectory feasibility. The precise alignment of the anchor bolts was confirmed prior to finalizing the position by aiming the laser head according to the planned trajectory.

### 2.5 Termination of animals

While still under anesthesia, the animals were euthanized according to the termination protocol with intravenous administration of sodium pentobarbital (>80 mg/kg) and was therefore in line with good clinical practice guidelines.

### 2.6 Statistical analysis

Descriptive analysis was performed on all conducted bone channels. In addition, a two-way analysis of variance test was performed for differences between anti-conical and cylindrical holes. All *p* values less than 0.05 were considered statistically significant.

## 3 Results

A total of 71 precision bone channels were cut in four pig specimens using a robot-guided laser. No signs of hemodynamic or respiratory irregularities were observed during anesthesia. A registration accuracy of less than 1.0 mm was achieved in all four pig specimens, and all channels were created according to the pre-planned cutting strategy.

**FIGURE 3:**
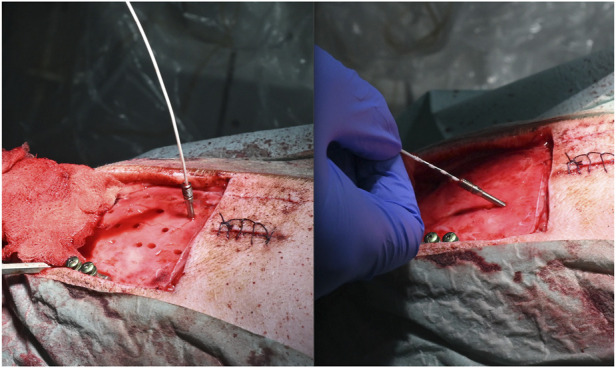
Possible different angulations. **(A)** Anchor bolt planned at 90° with inserted electrode and **(B)** anchor bold planned at 60° with inserted electrode.

**FIGURE 4:**
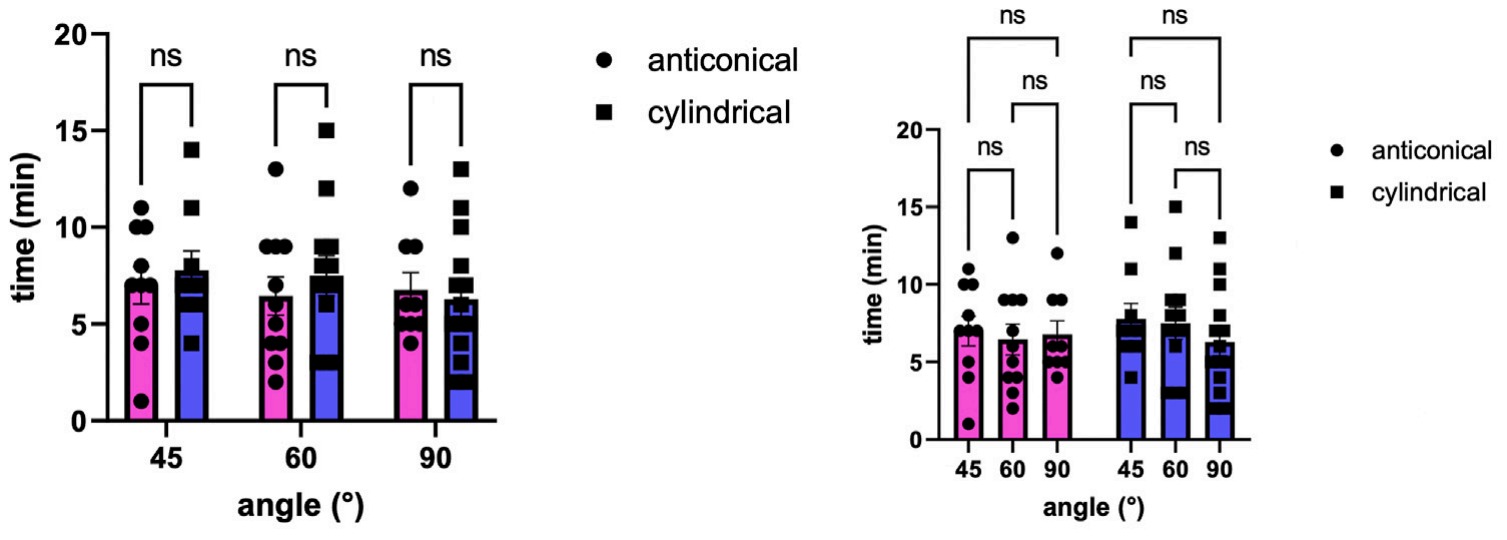
Time per hole by geometry and angulation.

The new cutting strategy showed no irregularities for either cylindrical (parallel walled; n = 38, 45° = 10, 60°14, 90° = 14) or anti-conical (walls widening by 2 degrees; n = 33, 45° = 11, 60° = 13, 90° = 9) bone channels. The entrance hole diameters ranged from 2.25 to 3.7 mm and the exit hole diameters ranged from 1.25 to 2.82 mm. The diameters were larger in holes angulated at either 45 or 60° compared with those angulated at 90° (Figure 3). No significant differences in angulation or cutting depth were found between the cylindrical and anti-conical cutting strategies. Moreover, there was no significant difference in the time per hole between anti-conical and cylindrical holes at different angulations (two-way analysis of variance; F (2, 59) = 0.3594; *p* > 0.05) (Figure 4).

**FIGURE 5:**
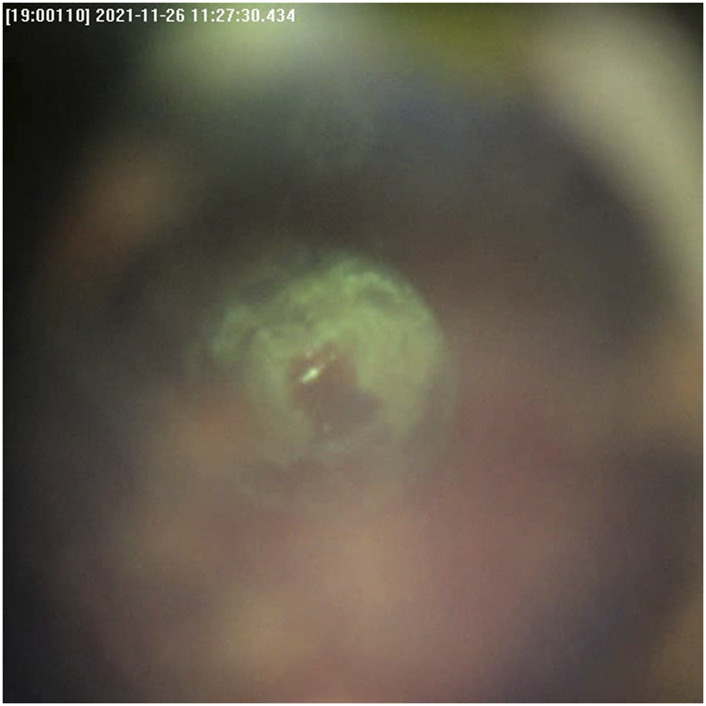
Cut-through detection with the live-feed coaxial camera system.

**FIGURE 6:**
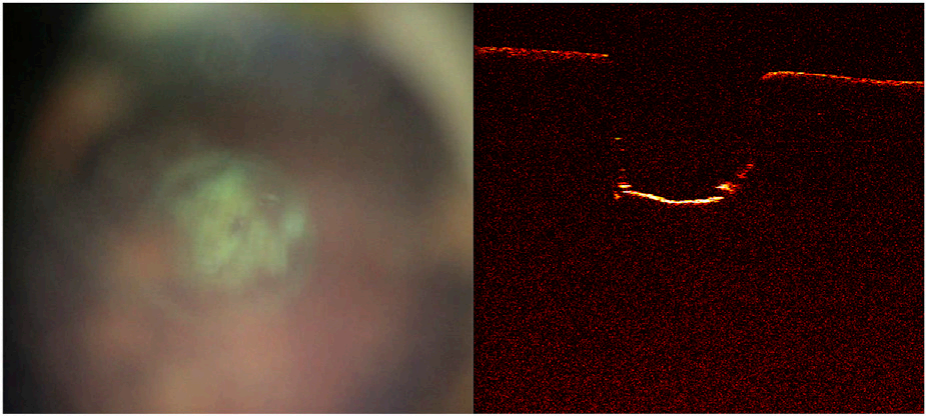
Cut-through not yet achieved as shown on the **(A)** live-feed coaxial camera system and **(B)** optical coherence tomography image. Further laser pulses are needed to achieve cut through.

The quality of the live video feed increased with the new built-in coaxial illumination (Figure 5). The updated live-feed coaxial camera system, along with the OCT signal, reliably detected cut-through (Figures 6A,B).

**FIGURE 7:**
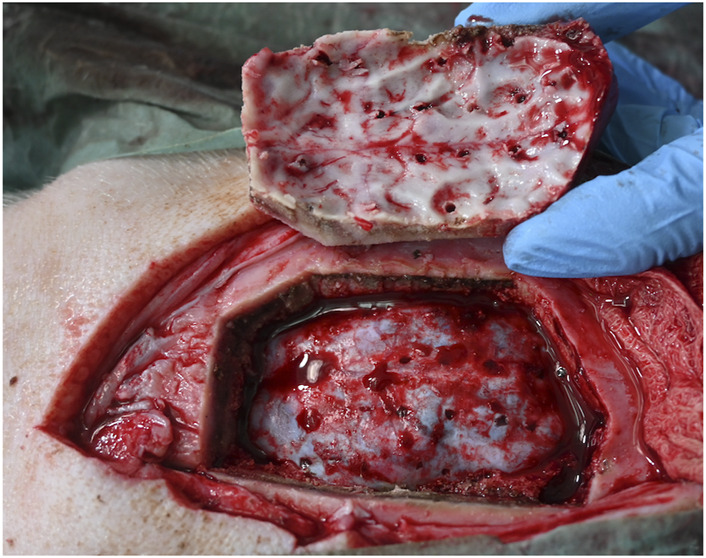
No unintended damage is observed in underlying tissue. Dura penetration can be seen only at the electrode insertion sites.

Anchor bolts and depth electrodes were implanted guided solely by the laser precision channels. Bolt insertion was achieved in all bone channels. For both anti-conical and cylindrical holes, haptic feedback during guide nut insertion confirmed a tight fit and was recorded in all cases, independent of angulation. No unintended damage to the cortex was detected after laser guided craniotomy (Figure 7).

## 4 Discussion

The updated hardware and software used in this study demonstrate the potential of this technology. This data may provide a baseline for further research as the first *in vivo* recovery animal study. This large *in vivo* non-recovery series supports the findings of previous studies and showed improvement in cut-through detection in more than 70 burr holes in the skull plate. As observed in previous studies, the laser leaves the ablated bone surface porous and biologically functional (Baek et al., 2015; Augello et al., 2018). However, inflowing liquids, either blood from porous bone or cerebrospinal fluid leakage, impede the cutting efficiency, as laser power is weakened or absorbed respectively before reaching the target (Winter et al., 2021). In addition, the live-feed coaxial camera system as well as the OCT signals were less reliant with inflowing liquids, and the laser was unable to penetrate deeper through the liquid. However, using the new cutting strategy the laser was able to sufficiently cut the bone channels, including the outlet edge, and depth electrodes were inserted with no resistance. Therefore, if liquid influx remains in balance with the working laser speed, cut-through detection is clear and evident in both the live video feed and OCT signals.

The cutting strategy used in previous trials includes a pin of bone in the center of the hole, which should be removed by a final high energetic “super-burst.” Consequently, as the remaining bone fragments could not be reliably removed, this strategy was replaced by the current one. The new strategy is based on a centric pilot shot followed by a surrounding hollow-cylinder, thereby shaping the contour of the hole. Once cut-through is confirmed, the outer section can be divided into multiple sub-sections for a selective ablation of the remaining lamella, while minimizing uncontrolled damage to the underlying tissues. The centric pilot shot serves as the primary information source for depth control, especially based on the OCT signal, which penetrates into the bone. From a clinical and risk viewpoint, this results in several benefits. The energy may be reduced towards the bottom of the hole to safely achieve cut-through. A centric pilot hole with a diameter of more than 1 mm guarantees an unobstructed pathway for electrode insertion. Any remaining bone lamella at the outlet profile would neither impair the insertion of the guide nut nor the subsequent insertion of the electrode. The hole can be independently verified by physically probing, such as with the tip of a monopolar or similar device. And the new cutting strategy proved to be faster than the previous one.

Previous studies on performing laser osteotomies included limited visual inspection options (Stübinger et al., 2009). However, we believe a visual inspection increases the safety in laser osteotomy performance. Hence, an upgrade to the coaxial camera with coaxial illumination was implemented and the hardware around the nozzle with cooling spray was adapted. Therefore, our visual inspection capability was improved compared with that of previous studies (Augello et al., 2018; Roessler et al., 2021, Winter et al., 2021), thereby improving the feasibility and credibility of cut-through detection. Further studies are planned in an *in-vivo* recovery setting following first in human studies for safe robot-driven laser craniotomy for stereoelectroencephalography procedures.

### 4.1 Limitations

As this was a non-recovery study, postoperative outcomes could not be evaluated. However, all planned trajectories were performed with no intraoperative anesthesiologic irregularities in this *in vivo* study. Furthermore, as no postoperative imaging was performed, the accuracy of the preplanned trajectories could not be evaluated.

## Conclusion

This new cutting strategy showed promising results in more than 70 precision angulated cylindrical and anti-conical bone channels in a large, *in vivo* non-recovery animal study. This study proved the feasibility and validity of OCT signals and especially the improved coaxial camera system for cut-through detection. Further studies are needed to evaluate the postoperative outcomes and accuracy of inserted electrodes with postoperative imaging.

## Data Availability

The raw data supporting the conclusion of this article will be made available by the authors, without undue reservation.
